# Implementation and experimental evaluation of Mega-voltage fan-beam CT using a linear accelerator

**DOI:** 10.1186/s13014-021-01862-x

**Published:** 2021-07-28

**Authors:** Hao Gong, Shengzhen Tao, Justin D. Gagneur, Wei Liu, Jiajian Shen, Cynthia H. McCollough, Yanle Hu, Shuai Leng

**Affiliations:** 1 Department of Radiology, Mayo Clinic, 200 First Street SW, Rochester, MN 55905 USA; 2 Department of Radiology, Mayo Clinic Arizona, 5881 East Mayo Blvd, Phoenix, AZ 85258 USA

**Keywords:** Dose calculation, Electron density, Mega-voltage CT, Radiation therapy

## Abstract

**Background:**

Mega-voltage fan-beam Computed Tomography (MV-FBCT) holds potential in accurate determination of relative electron density (RED) and proton stopping power ratio (SPR) but is not widely available.

**Objective:**

To demonstrate the feasibility of MV-FBCT using a medical linear accelerator (LINAC) with a 2.5 MV imaging beam, an electronic portal imaging device (EPID) and multileaf collimators (MLCs).

**Methods:**

MLCs were used to collimate MV beam along z direction to enable a 1 cm width fan-beam. Projection data were acquired within one gantry rotation and preprocessed with in-house developed artifact correction algorithms before the reconstruction. MV-FBCT data were acquired at two dose levels: 30 and 60 monitor units (MUs). A Catphan 604 phantom was used to evaluate basic image quality. A head-sized CIRS phantom with three configurations of tissue-mimicking inserts was scanned and MV-FBCT Hounsfield unit (HU) to RED calibration was established for each insert configuration using linear regression. The determination coefficient (${R}^{2}$) was used to gauge the accuracy of HU-RED calibration. Results were compared with baseline single-energy kilo-voltage treatment planning CT (TP-CT) HU-RED calibration which represented the current standard clinical practice.

**Results:**

The in-house artifact correction algorithms effectively suppressed ring artifact, cupping artifact, and CT number bias in MV-FBCT. Compared to TP-CT, MV-FBCT was able to improve the prediction accuracy of the HU-RED calibration curve for all three configurations of insert materials, with ${R}^{2}$ > 0.9994 and ${R}^{2}$ < 0.9990 for MV-FBCT and TP-CT HU-RED calibration curves of soft-tissue inserts, respectively. The measured mean CT numbers of blood-iodine mixture inserts in TP-CT drastically deviated from the fitted values but not in MV-FBCT. Reducing the radiation level from 60 to 30 MU did not decrease the prediction accuracy of the MV-FBCT HU-RED calibration curve.

**Conclusion:**

We demonstrated the feasibility of MV-FBCT and its potential in providing more accurate RED estimation.

## Background

Radiation therapy (RT) is one of the most common cancer treatment methods [1, 2]. It is minimally invasive and has the flexibility of optimizing radiation dose distributions conformal to treatment targets. The adoption of pre-treatment volumetric imaging and multileaf collimators (MLCs) in RT has enabled highly conformal treatment plans, which deliver lethal radiation dose to tumor while sparing organs-at-risk (OARs) surrounding the tumor [3]. Effective RT treatment relies on accurate calculation of radiation dose distribution within patients. Minimizing deviation between the calculated and delivered doses is always beneficial as it provides better estimation of doses delivered to the target and OARs and allows us to correlate radiation doses with treatment outcomes more accurately [4–6].

In clinical practice, relative electron density (RED) or proton stopping power ratio (SPR) is required to calculate photon or proton dose distribution, respectively [7–9]. These quantities can be obtained from CT numbers using a calibration curve established during the commissioning. CT number is a measure of linear attenuation coefficient which reflects a combined effect of Compton scattering ($\propto RED$), coherent scattering ($\propto {Z}^{1.86}$) and photoelectric ($\propto {Z}^{3.62}$) interaction mechanisms [8]. It depends not only on RED but also on chemical composition (or effective atomic number). Currently, treatment planning is based on treatment-planning CT (TP-CT) using kilovoltage X-ray beams. In the energy range of TP-CT, Compton scattering is the main interaction mechanism but photoelectric effect and coherent scattering are not negligible. Materials with different chemical compositions may have the same CT numbers but different REDs or proton SPRs (degeneracy effect). However, based on the calibration curve method, these materials are mapped to the same RED or proton SPR, causing errors in RED or proton SPR estimation.

Compared to photon therapy, proton therapy dose calculation is more sensitive to the degeneracy effect. As an extreme example, if we add 1 cm of bolus in the beam path proximal to the treatment target which is equivalent to overriding 1 cm of air (HU = − 1000) to water (HU = 0), it results in only 2–3% of reduction in calculated dose to the treatment target for photon therapy. This, however, can pull back the spread-out Bragg peak by 1 cm for proton therapy, causing significant reduction in calculated dose to the treatment target. The degeneracy effect, although small, cannot be neglected for proton therapy. Clinically, an extra margin (~ 3.5% of water equivalent depth) is usually used to compensate potential errors in proton SPR estimation, or proton range uncertainty.

To reduce proton range uncertainty, one possible solution is dual-energy CT (DE-CT). Another potential solution is MV-FBCT, which is less susceptible to the degeneracy effect (or chemical composition variation) compared to TP-CT. Of note, in our recent study about proton range uncertainty [10], we have demonstrated that: first, the HU to SPR calibration curve is comprised of two segments, i.e. HU to RED conversion and RED to proton SPR conversion; second, when x-ray photon energy is within kV range, the proton range uncertainty mainly resides in the segment of HU to RED conversion. As we move to MV-FBCT, contributions from the photoelectric interaction ($\propto {Z}^{3.62}$)/coherent scattering and the sensitivity to chemical composition variation drop substantially compared to TP-CT. As a result, MV-FBCT HU maintains a better linear relationship with RED compared to treatment-planning CT. Meanwhile, mapping of RED to proton SPR goes through a logarithm operation which significantly suppresses the effect of chemical composition variation. Therefore, a calibration curve mapping from MV-FBCT HU to proton SPR is supposed to be more robust to chemical composition variation and thus has the potential to reduce proton range uncertainty. In a previous study intended to use MVCT to reduce proton range uncertainty near large metal implants, Newhauser et al. demonstrated the superb linear relationship between MVCT HU and proton SPR [11].

As of today, DECT has been thoroughly investigated but MV-FBCT has not received sufficient attention. A practical challenge of MV-FBCT is its availability. Currently, MV-FBCT is only available on Tomotherapy and Accuray Radixact (the next generation of TomoTherapy) systems. Yet, MV-FBCT in these systems is primarily designed for the purpose of patient setup. Use of these systems for treatment planning may be good enough for photon therapy as it is quite insensitive to chemical change variation, but may not be sufficient for proton therapy. In addition, these systems may not be available to many cancer centers.

The purpose of this work was to develop a method to acquire MV-FBCT images using the MV imaging beam, EPID, and MLC of a LINAC to improve the availability of MV-FBCT for the purpose of treatment planning. In addition, we developed an in-house data preprocessing pipeline to correct MV-FBCT image artifacts and CT number deviation caused by defects in raw projection data. We also assessed image quality of MV-FBCT and evaluated the prediction accuracy of the MV-FBCT HU-RED calibration curve compared to TP-CT. Even though Varian TrueBeam LINAC was used to demonstrate the feasibility of MV-FBCT in this study, the proposed method may be extended to other LINACs as well. With increased availability of MV-FBCT, we may stimulate more activities in exploring the potential of using MV-FBCT to reduce proton range uncertainty.

## Methods

### Data acquisition

A standard TrueBeam LINAC (Varian Medical Systems, Palo Alto, California, USA) was used to acquire MV-FBCT, using a 2.5 MV imaging beam, a 43 × 43 cm^2^ EPID placed 1.5 m from the source. An MLC was collimated to an in-plane field-of-view (FOV) of 28.7 cm and a cross-plane width of 1.0 cm at the isocenter. The axial scan trajectory was used in data acquisition, with 306 projections per revolution. Projection data was acquired in a full rotation and MV-FBCT images were reconstructed using a standard FDK algorithm with the Hanning filter after correction of X-ray scatter, data truncation, beam-hardening, and ring-artifact. MV-FBCT data was acquired at two radiation dose levels of 30 and 60 MUs. A Catphan (Model 604, The Phantom Laboratory, Salem, New York, USA) was scanned to evaluate image quality. A head-sized CIRS phantom (Model 062 M, CIRS, Norfolk, Virginia, USA) with three configurations of tissue-mimicking CIRS inserts and Gammex inserts (Sun Nuclear, Melbourne, Florida, USA) was used to evaluate HU value accuracy. Table 1 listed parameters used in MV-FBCT data acquisition. Table 2 listed all insert materials in each configuration. For both phantoms, the reconstructed FOV was 20 cm in diameter, the slice thickness and increment were both 0.1 cm for the high resolution module of the Catphan phantom and both 0.3 cm for all other cases.

**Table 1:** The parameters of imaging geometry and scanning protocols in MV-FBCT

Peak X-ray energy	2.5 MeV
Source-to-isocenter distance	100 cm
Source-to-detector distance	150 cm
Detector matrix size	1280 × 1280
Detector pixel size	0.0336 cm
Scanning FOV (at iso-center)	28.6 cm
Width of MLC slit (at iso-center)	1.0 cm
Radiation dose (in delivered machine unit)	30 & 60 MU
Number of projection per revolution	306

**Table 2:** The configurations of insert materials

Config #1 (Gammex inserts)	Config #2 (CIRS low-density inserts)	Config #3 (CIRS bone inserts)
Adipose	Adipose	Bone 200 mg/cc
Brain	Breast 50/50	Bone 800 mg/cc
Blood + Iodine (2 mg/cc)	Liver	Bone 1000 mg/cc
Blood + Iodine (4 mg/cc)	Lung (Exhale)	Bone 1250 mg/cc
Blood	Lung (Inhale)	Bone 1500 mg/cc
Breast 50/50	Muscle	Bone 1750 mg/cc
Liver	Plastic water	
Solid water		

To demonstrate that MV-FBCT HU maintains a better linear relationship with RED compared to TP-CT HU, we also acquired TP-CT images of tissue-mimicking inserts using the same phantom setups on a commercial TP-CT simulator (Somatom Definition AS, Siemens Medical Solution USA, Malvern, PA, USA). Key imaging parameters used in TP-CT included 120 kVp, 200 mAs, 0.8 pitch, 30 cm reconstructed FOV, and 0.3 cm slice thickness and increment.

### Artifact correction in projection domain

An in-house preprocessing pipeline was implemented to suppress MV-FBCT image artifacts that degraded CT number accuracy. This pipeline included the correction for X-ray scatter, projection data truncation, beam-hardening, and ring-artifact. Methods of each correction step are introduced below.

#### Scatter correction

**Fig. 1:**
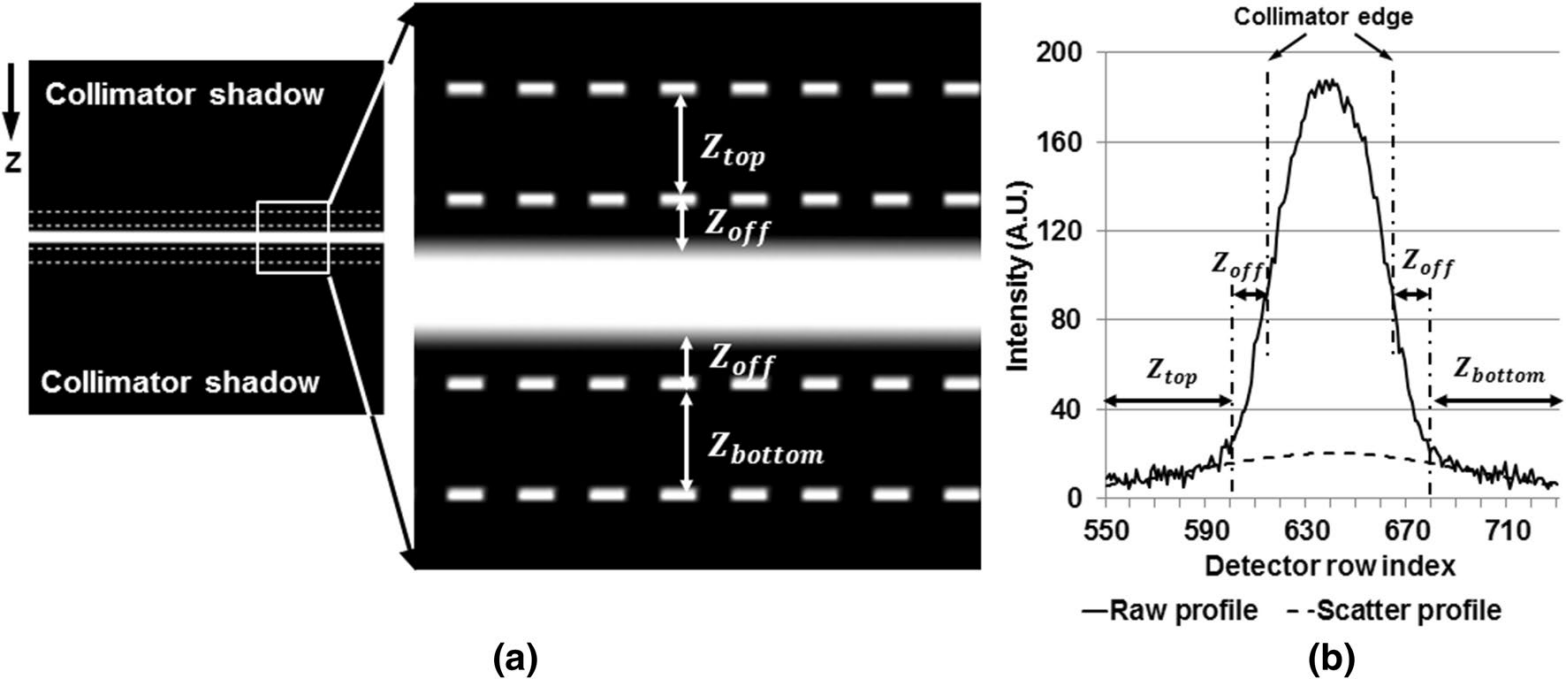
**a** Schematic illustration of region of support that was used in scatter correction. **b** The profiles of the measured detector signal (denoted as “Raw profile”) and the estimated scatter signal (denoted as “Scatter profile”). The profiles were extracted from the central detector column. ${{\varvec{Z}}}_{{\varvec{o}}{\varvec{f}}{\varvec{f}}}$ denotes the stand-off region. ${{\varvec{Z}}}_{{\varvec{t}}{\varvec{o}}{\varvec{p}}}$ and ${{\varvec{Z}}}_{{\varvec{b}}{\varvec{o}}{\varvec{t}}{\varvec{t}}{\varvec{o}}{\varvec{m}}}$ denote the region of support where the measured detector signal was used as scatter fluence

The method of scatter correction used in this study was similar to the one in Reference [12], with the assumption that the detector signal behind the collimator blades was fully induced by X-ray scatter. Specifically, the detector signal measured from collimator shadow was directly used to estimate the scatter fluence outside the primary beam. Measurement was performed in the region of support ${Z}_{top}$ and ${Z}_{bottom}$ (both covered 50 detector rows, i.e. 16.8 mm) (Fig. 1), which was separated from the collimator edges by a small stand-off region (${Z}_{off}$ = 18 detector rows, i.e. 6.05 mm) to avoid influence of potential penumbra and extra-focal radiation. Then, 1D scatter profile under the collimator slit was estimated by fitting single Gaussian kernel (Eq. (1)) along each detector column:

1 $${\mathrm{S}}_{\mathrm{est}} =\mathrm{ a}\cdot \mathrm{exp}\left(-{\left(\frac{{\mathrm{S}}_{\mathrm{meas}}-\mathrm{b}}{\mathrm{c}}\right)}^{2}\right)$$

where ${S}_{est}$ denotes the estimated 1D scatter profile under the collimator slit at a given detector column, ${S}_{meas}$ denotes the measured scatter profile from collimator shadow, and $a$, $b$ and $c$ denote the kernel parameters that were calculated during fitting. The 1D scatter profile of all detector column formed 2D scatter fluence. Then, the 2D scatter fluence was further smoothed by applying a 2D median filter (with a square kernel of 13 × 13 detector pixels). Finally, the estimated scatter fluence was subtracted from the raw projection measured at each view, to suppress scatter artifact.

#### Projection data truncation correction

In MV-FBCT, the scanning FOV did not fully cover the treatment couch. The obvious attenuation of treatment couch outside of FOV caused truncation artifact which contributed to non-uniform CT number bias across the reconstructed images. The reason is briefly explained as follows. As part of the couch was outside the FOV of MV-FBCT, the acquired projection data suffered from truncation (i.e. no measured projection data along rays outside of the FOV) and thus no longer met the data sufficiency condition for the theoretically-exact CT image reconstruction [13–16]. As a result, non-uniform CT number bias was present across the reconstructed FOV, with CT number gradually decreased towards the center of the FOV [15–18]. To correct the truncation artifact, attenuation of treatment couch was measured, without placing phantoms in the scanning FOV. Scatter correction was also conducted in each view as described in Sec. 2.2.1. There existed slight random view offsets (roughly range from 0.1° to 0.5°) between phantom scans and patient couch scan, due to the finite tolerance of gantry rotation. Such view offset induced obvious CT number bias. To overcome this problem, an angular shape-preserving piece-wise cubic interpolation was employed to estimate the patient couch attenuation at exactly the same views in phantom scans. Briefly, the expected signal of each detector pixel at the missing views was interpolated from the measured signal of the same detector pixel across all views acquired in the patient couch scan. Finally, view-wise subtraction was used to remove patient couch attenuation from the measured phantom attenuation.

#### Beam-hardening correction

**Fig. 2:**
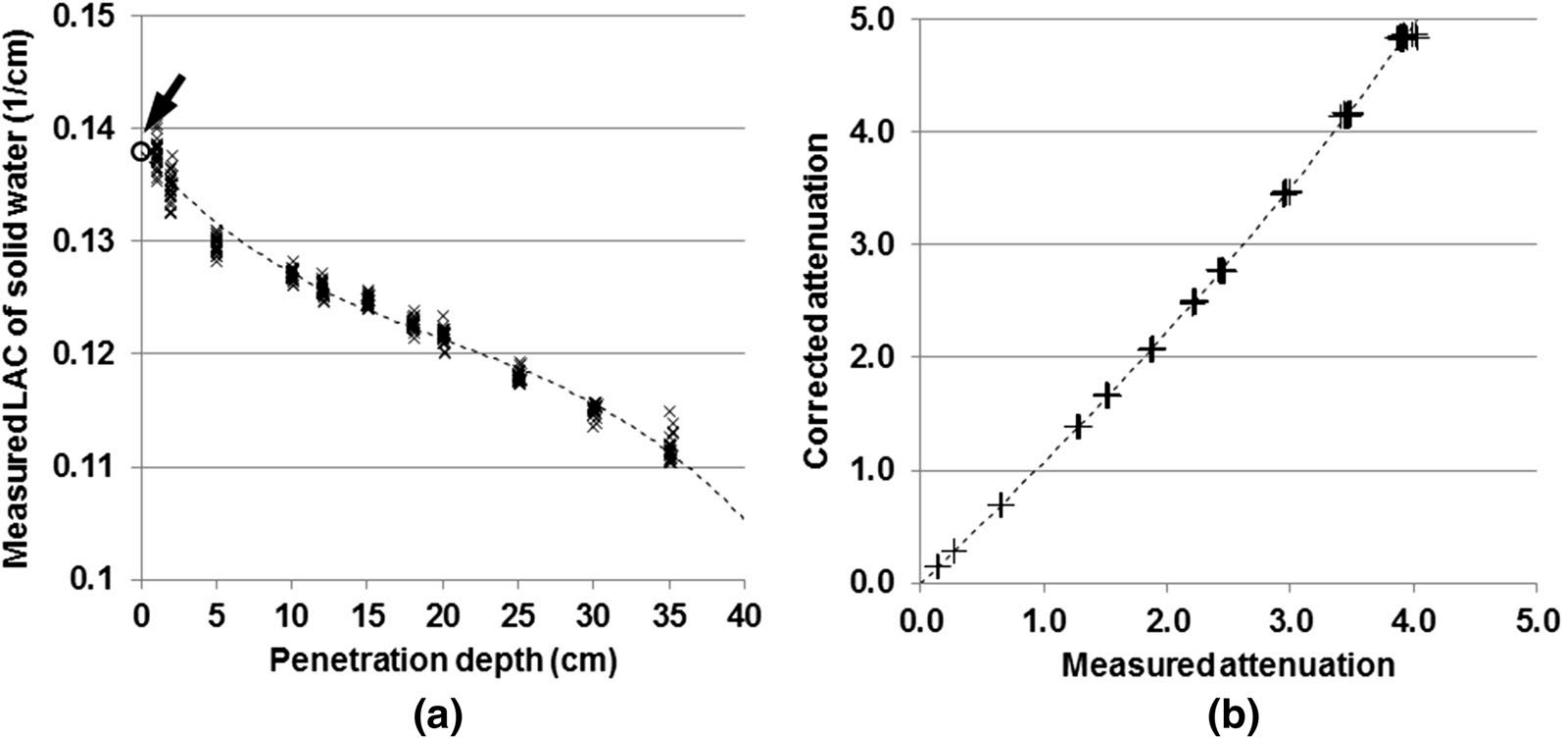
**a** The 3-degree polynomial fitting between the measured linear attenuation coefficient (LAC) and the corresponding penetration depth. The arrow indicates the estimated un-hardened LAC (i.e. 0.1380 1/cm) at 0 cm penetration depth. **b** The 3-degree polynomial fitting between the measured solid water attenuation and the corrected attenuation. The formula of the polynomial model is shown in Eq. (2)

We acquired the projections of a set of solid water slabs with various thickness ($n = 11$, thickness ranged from 1 to 35 cm) from MV-FBCT, with the primary beam being perpendicular to the water slabs and patient bed. The correction of scatter and projection data truncation was conducted before further processing. Then, the hardened linear attenuation coefficient (LAC) at each solid water thickness was measured by taking the logarithm of the ratio between the measured solid water attenuation and air projection. A 3-degree polynomial regression model was obtained by fitting the hardened LAC over the penetration depth (Fig. 2). The un-hardened LAC (0.1380 1/cm) of solid water was estimated by extrapolating hardened LAC at 0 thickness of solid water using the regression model. The un-hardened solid water attenuation was then calculated by multiplying the un-hardened LAC with varying penetration depth. Further, a secondary 3-degree polynomial regression model was built by fitting the measured solid water attenuation ${P}_{meas}$ over the estimated un-hardened solid water attenuation ${P}_{correct}$:

2 $${P}_{correct}=0.00754\cdot {P}_{meas}^{3}+ 0.0170\cdot {P}_{meas}^{2}+ 1.054\cdot {P}_{meas}- 0.0067$$

All measured projection data were processed with Eq. (2) to correct beam hardening effect.

#### Ring artifact correction

**Fig. 3:**
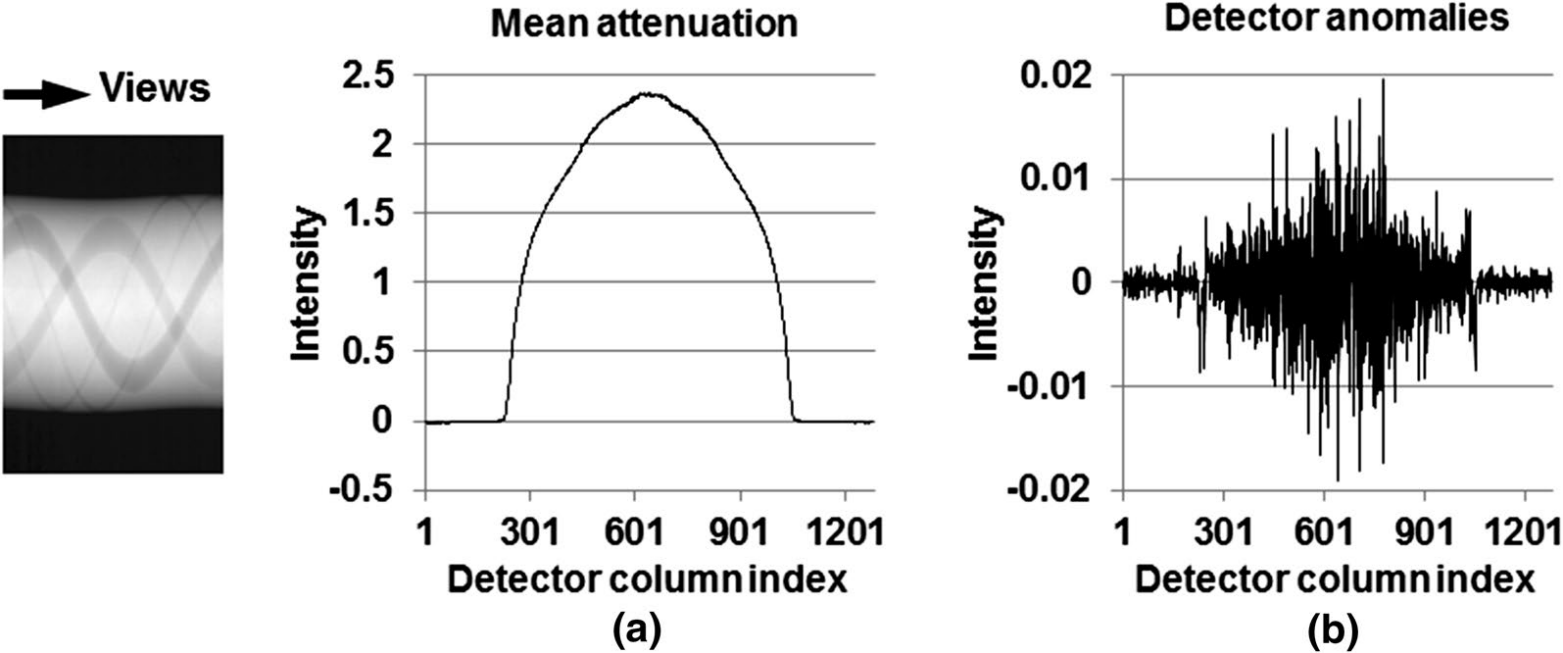
The example profiles of mean attenuation (**a**) and detector anomalies (**b**) from the sinogram shown in the inset. The mean attenuation was calculated by averaging the measured attenuation along the view direction. The magnitude of detector anomalies was the difference between the mean attenuation and the smoothed mean attenuation

Ring artifact correction was carried out in the sinogram extracted from each detector row, after the corrections of X-ray scatter, projection truncation, and beam-hardening. For each sinogram, the mean attenuation profile was calculated by averaging the detector signal across all views (Fig. 3a). Then, the mean attenuation profile was smoothed using a moving-average filter with a 7 × 1 kernel. The detector anomalies were manifested as the difference between the smoothed and un-smoothed mean attenuation (Fig. 3b). Finally, the detector anomalies were subtracted across all views in each sinogram, to suppress the ring artifact.

### Evaluation methods

We compared the image quality of Catphan phantom before and after applying the in-house artifact corrections. The image quality was evaluated in terms of image artifacts, spatial resolution, CT number bias, uniformity and contrast-to-noise-ratio (CNR). Further, we measured the mean CT numbers of varying Gammex and CIRS inserts in MV-FBCT and TP-CT images, respectively. The accuracy of HU-RED calibration was separately evaluated in the three configurations of insert materials with known REDs (Table 2). Specifically, linear regression between mean CT numbers and REDs was conducted with the interception term fixed at 1.0. The determination coefficient (i.e. ${R}^{2}$) was used to gauge the accuracy of correlation between CT number and RED. The results acquired from TP-CT were used as the baseline to be compared with.

## Results

### The effects of artifact correction

**Fig. 4:**
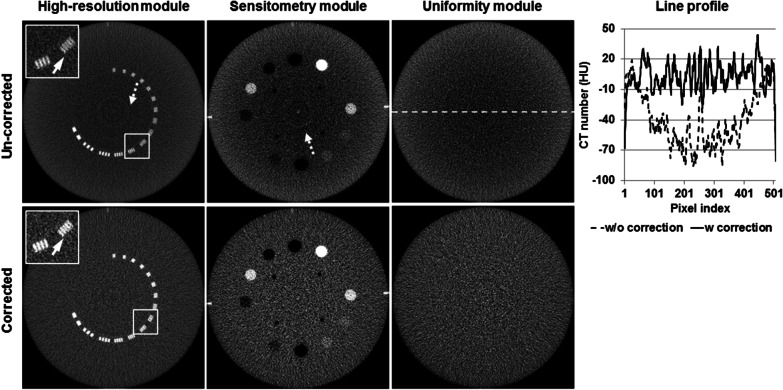
The mega-voltage fan-beam CT images of Catphan phantom before and after applying the in-house artifact correction. The corresponding projection data was acquired with 60 MU. The image thickness was 1 mm, 3 mm, and 3 mm for high-resolution, sensitometry, and uniformity modules, respectively. For high-resolution module, the display window was fixed at W/L: 800/100 HU. For the other two modules, the display window was at W/L: 400/40 HU. The solid arrows indicate the resolution test gauge at 6 line pairs per cm. The dashed arrows indicate the ring artifact. The dashed line indicates the location where the line profiles were extracted

Without preprocessing, MV-FBCT images presented significant cupping artifact and ring artifact (e.g. Catphan images in Fig. 4). Cupping artifact induced non-uniform CT number bias across the entire images. The maximal CT number bias was approximately 70 HU in the uniformity module before artifact correction was applied (see the line profiles in Fig. 4). The in-house artifact correction successfully suppressed image artifacts, and maintained image uniformity and spatial resolution (e.g. the maximal resolution 6 ± 0.5 line pairs/cm with 1 mm image thickness). Image uniformity was evaluated with the uniformity module of Catphan phantom, comparing the mean CT number measured from one central region-of-interests (ROI) and four peripheral ROIs (Table 3). Contrast-to-noise-ratio (CNR) was measured with Catphan sensitometry module (Table 4).

**Table 3:** Evaluation of image uniformity with Catphan uniformity module: mean CT number ± standard error at each region-of-interest (ROI) *

Dose level	30 MU	60 MU
Central ROI	1.7 ± 2.0 HU	0.6 ± 1.2 HU
Peripheral ROI #1	1.5 ± 1.6 HU	− 1.0 ± 0.9 HU
Peripheral ROI #2	1.3 ± 1.6 HU	− 0.2 ± 0.9 HU
Peripheral ROI #3	− 0.4 ± 1.5 HU	− 0.4 ± 1.0 HU
Peripheral ROI #4	− 1.4 ± 1.6 HU	− 1.1 ± 1.0 HU

^*^In-plane ROI diameter—2.3 cm; slice thickness—0.3 cm

**Table 4:** Evaluation of contrast-noise-ratio (CNR) with Catphan sensitometry module*

	Dose level	30 MU	60 MU
	Insert #1	3.7	6.4
	Insert #2	1.2	2.2
	Insert #3	0.2	0.4
	Insert #4	0.2	0.5
	Insert #5	0.3	0.6
	Insert #6	0.7	1.1
	Insert #7	1.2	2.2
	Insert #8	1.0	1.8

^*^For CNR measurement at each insert, the in-plane ROIs were placed at the center of each insert and the immediate background region. ROI diameter—0.8 cm; slice thickness—0.3 cm. The inset illustrates the indices of inserts

### Correlation analyses

**Fig. 5:**
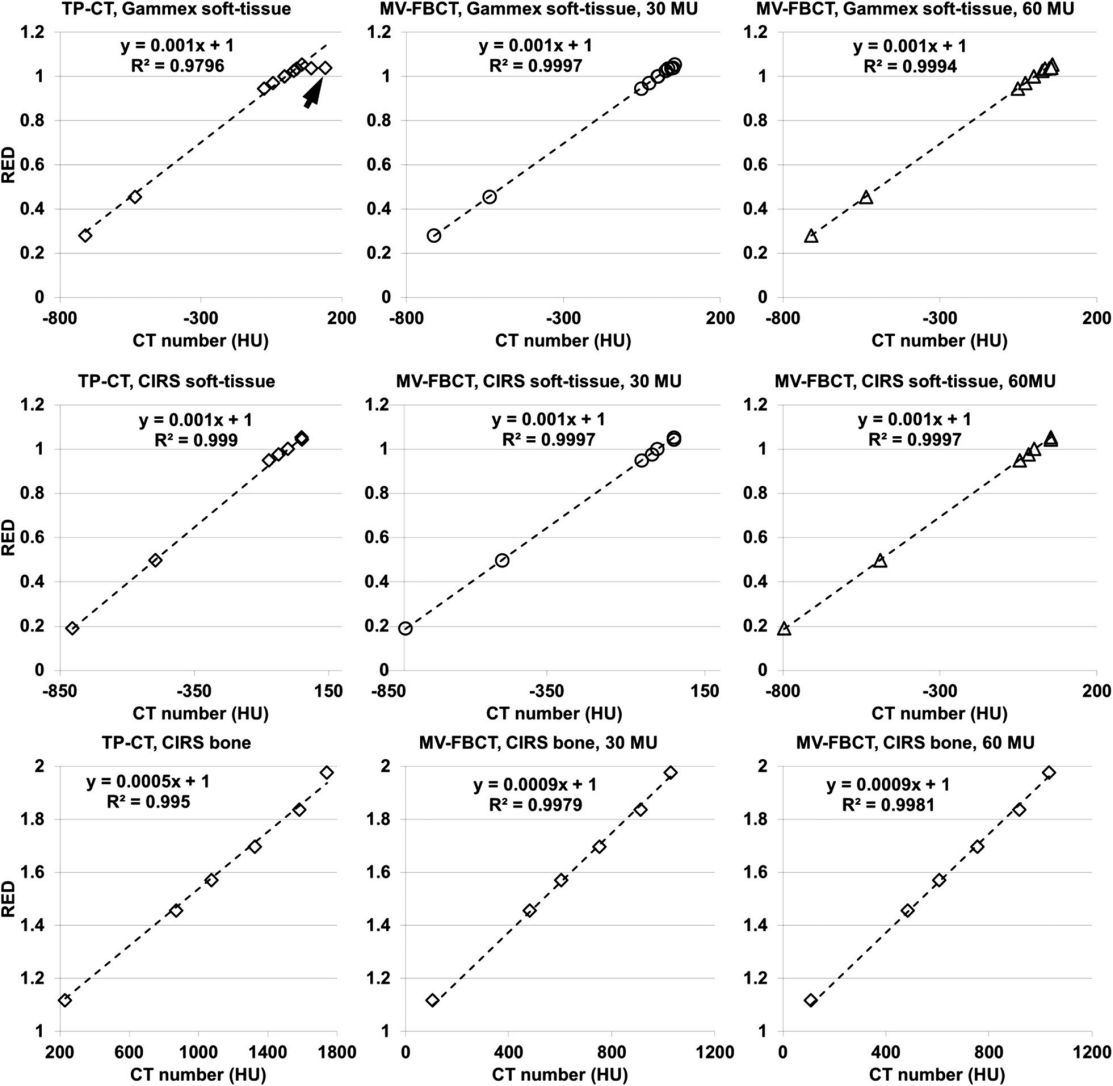
The linear regression between relative electron densities (RED) and mean CT numbers in clinical CT (TP-CT, with routine radiation dose) and mega-voltage fan-beam CT (MV-FBCT, with 30 MU and 60 MU). The Gammex soft-tissue inserts (top charts) include adipose, brain, breast, blood, liver, and solid water. The CIRS soft-tissue inserts (bottom charts) include lung (inhale), lung (exhale), adipose, breast, solid water, muscle, and liver. For bone inserts, the corresponding mass densities of ranged from 200 to 1750 mg/cc. The formula of linear regression and the determination coefficient are presented next to the dashed trendline. The solid arrow indicates the points with respect to the blood-iodine mixture inserts

Compared to TP-CT, MV-FBCT yielded relatively stronger linear correlation between mean CT numbers and REDs of all insert materials. For soft-tissue inserts, the value of ${R}^{2}$ was $\le$ 0.9990 in TP-CT but $\ge$ 0.9994 in MV-FBCT (Fig. 5). Moreover, we observed that the measured mean CT numbers of Gammex blood-iodine mixture inserts in TP-CT drastically deviated from the fitted linear trendline (see the top left chart in Fig. 5) but not in MV-FBCT. This phenomenon is further discussed in Sec.4. For bone inserts, the value of ${R}^{2}$ was 0.9950 in TP-CT but $\ge$ 0.9979 in MV-FBCT, which may be attributed to less residual beam-hardening artifacts in MV-FBCT images. Further, the accuracy of linear correlation between CT numbers and REDs did not decrease at the lower radiation dose level (30 MU) in MV-FBCT (see the charts in the middle column of Fig. 5).

## Discussion

In this study, we implemented MV-FBCT on a commercial LINAC at our institution, using portal imaging functionality and MLC. Then, we developed in-house data preprocessing methods to correct cupping artifact (caused by beam hardening effect and scatter) and CT number bias in MV-FBCT images. Finally, we evaluated the accuracy of HU-RED calibration and compared to the standard clinical treatment planning CT, using three experimental configurations of different insert materials commonly seen in radiation therapy.

In the evaluation of HU-RED calibration, two Gammex blood-iodine mixture inserts (RED: 1.037 and 1.039) were included to mimic contrast-enhanced soft-tissue (e.g. Thyroid). The corresponding CT numbers from TP-CT obviously deviated from the extrapolated values according to the fitted curve (Fig. 5), and thus additional calibration would be necessary for these materials. This phenomenon was caused by the change of effective atomic number of different materials, which influenced the proportion of Compton scattering and photoelectric effects in TP-CT [19]. Further, other researchers have noted that TP-CT could result in ambiguity in HU-RED calibration curves over the similar RED range (approximately around RED 1.0) [20, 21]. In contrary, MV-FBCT could be employed to avoid such ambiguity and thus further improve calibration accuracy. Moreover, we observed ${R}^{2}$ value for bone inserts was slightly lower compared to that for soft-tissue inserts, indicating that a stronger beam-hardening correction may be needed for bone inserts in both TP-CT and MV-FBCT. Nevertheless, our experimental results indicated that MV-FBCT yielded stronger linear correlation between CT number and RED than TP-CT, and provided the potential to use single HU-RED calibration curve for all materials.

In this work, there was no flattering filter with the 2.5 MV beam, and thus the beam-hardening effect would be similar to that in MV-CBCT. Of note, the flattening filter could be used to further suppress beam-hardening artifact, which would be an interesting direction in a follow-up study. Due to larger collimation, MV-CBCT would yield much stronger scatter-to-signal ratio than MV-FBCT. Given the same incident dose, the scatter-corrected MV-CBCT image would tend to present stronger noise compared to MV-FBCT. If one aims to achieve the same image noise level, MV-CBCT would eventually induce higher radiation dose than MV-FBCT. In addition, our current scatter correction method may not be suitable for MV-CBCT, since the accuracy of the estimated scattering signal can decrease as less collimator shadow would be available. This is similar to what was already well-known in kV-CBCT [12]. To achieve better outcome, more advanced methods are likely needed for MV-CBCT scatter correction, e.g. the alternating pulse sequence based direct measurement [22]. Furthermore, MV-FBCT would greatly suppress the beam-hardening and metal implant artifacts, especially for the highly-attenuating materials. In contrary, these artifacts can still obviously corrupt DE-CT images, which degrades DE-CT number accuracy [23]. DE-CT has been investigated in a great extent, while MV-FBCT has not been thoroughly investigated.

We acknowledge several limitations in this preliminary study. First, the presented MV-FBCT only provides a sufficient scanning FOV for head/neck scans (28.7 cm). Truncation caused by patient table was corrected by subtracting projection from pre-scanned table. When MV-FBCT is applied to larger phantoms or body parts, it would be necessary to update the artifact correction methods to address additional projection truncation induced by larger subject size. Second, we did not assess MV-FBCT image quality and HU-RED calibration using dynamic phantoms. In practice, the movement of anatomical structure (e.g. heart and lung) can induce extra CT number bias. This is not a main concern for head and neck applications (the main potential applications due to limited FOV of the MV-FBCT) where there is relatively less patient motion. However, additional motion artifact correction would be mandatory, if MV-FBCT is applied in RT to thorax region. Third, we have not investigated the lowest imaging dose level that would be sufficient for dose calculation using MV-FBCT, neither the actual accuracy of dose calculation over anatomical structures. This study mainly focused on the feasibility of performing MV-FBCT using a commercial LINAC. Further studies are warranted to determine the lowest imaging dose and the accuracy of dose calculation over anatomic structures. Image acquisition time is also a limitation of the proposed method of using a LINAC to acquire MV-FBCT images. Since the MV-FBCT only covers 1 cm width in the superior-inferior (S/I) direction per gantry rotation (typically 1 min), MV-FBCT image acquisition time can be significantly longer compared to MV-CBCT or traditional TP-CT simulation for the same coverage. To minimize impact of slow image acquisition, it is recommended to acquire MV-FBCT only around the target area for the purpose of dose calculation and plan optimization. The larger scan range for patient setup and contouring of the entire organs-at-risk will be achieved using traditional TP-CT simulation. Another limitation is imaging dose. In this study, we focused on the feasibility investigation, instead of imaging dose. The dose used in this study is higher compared to kV CT simulation. Future studies focusing on minimizing imaging dose while maintaining image quality are warranted. Given the fact that MV-FBCT has long acquisition time and additional dose, use of MV-FBCT requires careful evaluation of risks and benefits. It may not be necessary for every disease sites. But for certain cases where critical structures (e.g. brain stem) are very close to the treatment target, use of MV-FBCT may become justifiable.

## Conclusions

We successfully implemented portal MV-FBCT imaging on a TrueBeam® LINAC that was originally equipped with on-board kV-CBCT. Our in-house artifact correction methods suppressed CT number bias induced by image artifacts. Finally, this in-house-implemented MV-FBCT may provide an accurate estimation of electron density distribution, potentially leading to accurate dose calculation for treatment plans in proton therapy.

## Data Availability

The datasets used and/or analyzed during the current study are available from the corresponding author on reasonable request.

## Abbreviations

EPID: Electronic portal imaging device
FBCT: Fan-beam CT
FOV: Field-of-view
HU: Hounsfield unit
LINAC: Linear accelerator
LAC: Linear-attenuation-coefficient
MLCs: Mutlileaf collimators
MU: Monitor unit
MVCT: Mega-voltage CT
MV-CBCT: Mega-voltage cone-beam CT
MV-FBCT: Mega-voltage fan-beam CT
OAR: Organ-at-risk
RED: Relative electron density
RT: Radiation therapy
SPR: Stopping power ratio
TP-CT: Treatment-planning Computed Tomography
